# Breaking barriers in electrochemical biosensing using bioinspired peptide and phage probes

**DOI:** 10.1007/s00216-024-05294-w

**Published:** 2024-04-19

**Authors:** Susana Campuzano, María Pedrero, Rodrigo Barderas, José M. Pingarrón

**Affiliations:** 1 Departamento de Química Analítica, Facultad de CC. Químicas, Universidad Complutense de Madrid, Pza. de las Ciencias 2, Madrid, 28040 Spain; 2 Chronic Disease Programme, UFIEC, Instituto de Salud Carlos III, Majadahonda, Madrid 28220 Spain; 3 CIBER of Frailty and Healthy Aging (CIBERFES), Madrid, Spain

**Keywords:** Electrochemical biosensing, Peptides, Phage probes, Phage display, Mimotopes

## Abstract

Electrochemical biosensing continues to advance tirelessly, overcoming barriers that have kept it from leaving research laboratories for many years. Among them, its compromised performance in complex biological matrices due to fouling or receptor stability issues, the limitations in determining toxic and small analytes, and its use, conditioned to the commercial availability of commercial receptors and the exploration of natural molecular interactions, deserved to be highlighted. To address these challenges, in addition to the intrinsic properties of electrochemical biosensing, its coupling with biomimetic materials has played a fundamental role, among which bioinspired phage and peptide probes stand out. The versatility in design and employment of these probes has opened an unimaginable plethora of possibilities for electrochemical biosensing, improving their performance far beyond the development of highly sensitive and selective devices. The state of the art offers robust electroanalytical biotools, capable of operating in complex samples and with exciting opportunities to discover and determine targets regardless of their toxicity and size, the commercial availability of bioreceptors, and prior knowledge of molecular interactions. With all this in mind, this review offers a panoramic, novel, and updated vision of both the tremendous advances and opportunities offered by the combination of electrochemical biosensors with bioinspired phage and peptide probes and the challenges and research efforts that are envisioned in the immediate future.

**Graphical Abstract:**

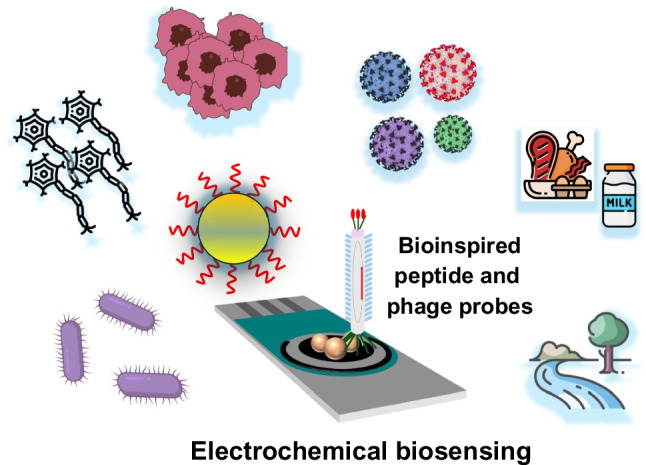

## Introduction

We are particularly excited to prepare this review and especially grateful to the Guest editors of this Topical Collection (Dr. Elena Benito-Peña and Prof. Guillermo Orellana) in honor of Prof. Mª Cruz Moreno-Bondi, who we consider a great University Professor and an outstanding researcher. She was our co-worker in the Analytical Chemistry Department (Chemistry Faculty, Universidad Complutense de Madrid), but also a great friend. We thought we could highlight a minimal part of her recent work showing her interest in the analytical applications of biomimetic recognition elements [1, 2], of bacteriophages in sensors development [3], or in phage display-based selection of recognition elements for biosensors [4], and even also describing a mimotope-based competitive immunoassay for the determination of a fungal toxin [5]. To do that, we have reviewed recent approaches using biomimetic functional materials in our field of work, electrochemical biosensing.

Biosensors and electrochemical biosensing continue to advance relentlessly overcoming barriers that were once perceived as obstacles to their transition from the research laboratory to the market. In these achievements, in addition to their intrinsic properties, their coupling with biomimicking materials has played a fundamental role. In this context, the opportunities imparted by bioinspired peptide and phage probes deserve to be highlighted. The versatility of these materials to be designed and employed has opened an unimaginable and exciting plethora of possibilities for electrochemical sensing and biosensing, improving its performance far beyond the development of sensitive and selective devices, allowing their evolution towards antibiofouling, trustworthy, robust, and easy-to-use devices able to provide easy-to-interpret results. These evolved devices show potential not only to determine targets independent of their toxicity and size, but also to discover new targets beyond commercial bioreceptors and natural and known molecular interactions.

**Fig. 1:**
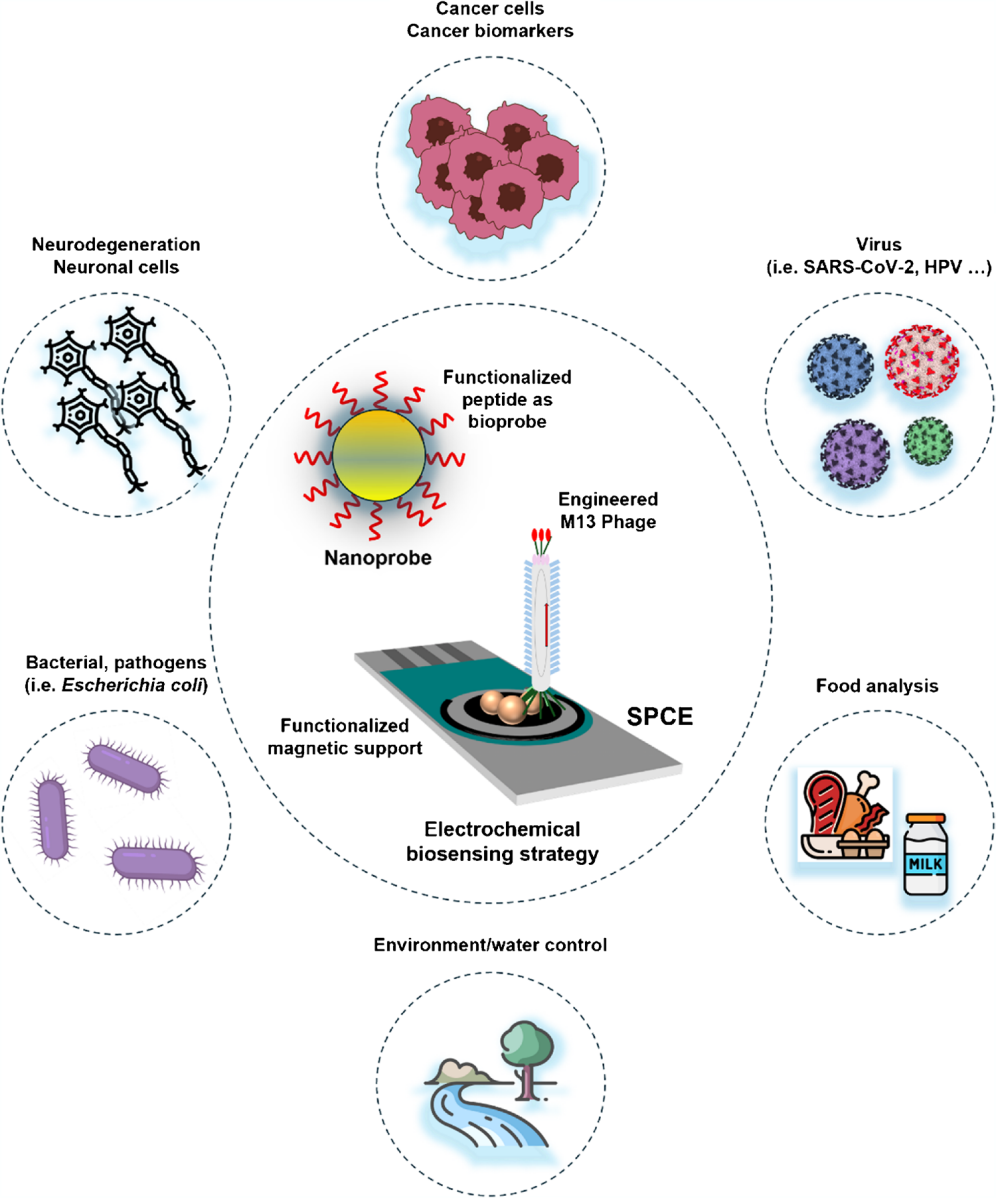
Schematic of the recent advances reviewed in this manuscript regarding the different applications of electrochemical biosensing involving peptide and phage probes. Different methods developed in the last 2 years involving peptide bioprobes (see Table 1) or phage bioprobes (see Table 2) are discussed in the manuscript. The versatility, pros and cons of peptide, and phage bioprobes in electrochemical biosensing are also discussed (see Table 3)

With all this in mind, this review aims to draw the current scenario outlined by the progress in the last 2 years in electrochemical biosensing involving peptide and phage probes (see Fig. 1). First, the intrinsic advantages of biomimetic receptors and electrochemical sensors and biosensors are briefly introduced, followed by a panoramic, novel, and updated view of the tremendous advances and opportunities provided by the combination of electrochemical biosensors with bioinspired phage and peptide probes to end with a somewhat more personal perspective on the message to keep and the challenges and research endeavors in the immediate future.

**Table 1:** Electrochemical biosensing methodologies reported since 2022 using peptide probes

Electrode	Peptide type/role	Fundamentals	Detection technique	Target analyte	Analytical characteristics	Application	Ref.
PWEs	Antimicrobial peptide Magainin I/biorecognition element	Direct affinity reaction at a UCNPs@SiO_2_@Ag/C-g-C_3_N_4_/PWE	PEC	*E. coli* O157:H7	L.R.: 5−5 × 10^6^ CFU mL^−1^ LOD: 2 CFU mL^−1^	Inoculated pork, cabbage, and milk samples	[32]
GCE	Multifunctional peptide (anchoring, antifouling, and two different recognizing branches)/biorecognition element	Direct affinity reaction at AuNPs/PEDOT/GCE modified with the multifunctional peptide	DPV ([Fe(CN)_6_]^3−/4−^)	IgG	L.R.: 0.1 pg mL^−1^−0.1 µg mL^−1^	Clinical serum samples	[33]
GCE	Antifouling peptide conjugated with recognizing DNA probes/modifier of another probe	Direct affinity reaction at AuNPs/PEDOT/GCE modified with streptavidin and biotinylated peptide-DNA conjugates	DPV ([Fe(CN)_6_]^3−/4−^)	CA125	L.R.: 0.01−1000 U mL^−1^ LOD: 3.0 mU mL^−1^	Clinical serum samples	[34]
AuE	Multifunctional peptide (anchoring, doping, antifouling, and recognizing functions)/biorecognition element	Direct affinity reaction at a AuE modified with the multifunctional peptide by self-assembly and PEDOT by electrodeposition	DPV ([Fe(CN)_6_]^3−/4−^)	MCF-7 cells	LOD: 17 cells mL^−1^	Spiked human blood	[35]
GCE	Multifunctional peptide/enzyme substrate	Cleaved of the multifunctional and dual biotinylated thiolated peptide immobilized on a Strep/PEG/PEDOT/GCE and MB/DNA/AuNRs for signal amplification	DPV (MB)	PSA	L.R.: 0.10 pg mL^−1^−10.0 ng mL^−1^ LOD: 0.035 pg mL^−1^	Human serum	[36]
AuE	Peptide derived from the docking motif of ELK-1/biorecognition reaction	Direct affinity reaction at an AuE modified with the peptide chemisorbed through its cysteine at N-terminus	EIS ([Fe(CN)_6_]^3−/4−^)	ERK2 kinase	L.R.: 0.5–2.0 μM LOD: 0.35 μM	–	[37]
Pyrolyzed carbon electrode	Switching peptide/signaling element	One-step immunoassay and displacement of Fc-labelled switching peptide in the presence of the target antigen	DPV (Fc)	hHBsAg	D.R.: 10−10^4^ nM LOD: 17 ng mL^−1^	s–	[38]
AuE	Oxidase-mimicking Cu^2+^-peptides/nanolabels	Sandwich immunosensor using AuNPs/peptide-Cu^2+^ conjugates the nanolabels	CV (oxygen reduction)	PSA	D.R.: 0.001–0.50 ng mL^−1^ LOD: 0.40 pg mL^−1^	Clinical serum samples	[27]
GCE	Metalloenzyme-mimicking Zn^2+^-peptides/bioreceptor	Direct detection at a GCE modified with a NiCo_2_O_4_-PAMAM-peptide composite and Nafion	SWV (PNP)	OPs (methyl paraoxon and ethyl paraoxon)	LOD: 0.08 μM (methyl paraoxon) and 0.16 µM (ethyl paraoxon)	Spiked *Brassica Chinensis L.*, tomatoes, and broccoli	[28]
GCE	Antifouling and antihydrolysis cyclic peptide/electrode modifier	Direct affinity reaction at a AuNPs/PEDOT/GCE modified with the cyclic peptide by self-assembly and ACE2 using EDC/NHS chemistry	DPV ([Fe(CN)_6_]^3−/4−^)	RBD of SARS-CoV-2	L.R.: 1.0 pg mL^−1^−100.0 ng mL^−1^ LOD: 0.45 pg mL^−1^	Spiked human blood	[24]
GCE	D‑amino acid-based antifouling peptides/electrode modifier	Direct immunosensing at a AuNPs/PEDOT/GCE and a specific antibody	DPV ([Fe(CN)_6_]^3−/4−^)	IgM	L.R.: 100 pg mL^−1^−1.0 μg mL^−1^ LOD: 37 pg mL^−1^	Clinical serum samples	[39]
GCE	Antifouling zwitterionic peptide hydrogel/electrode modifier	Direct affinity reaction at a GCE modified with PEDOT, the zwitterionic peptide, and a specific antibody	DPV ([Fe(CN)_6_]^3−/4−^)	PSA	L.R.: 0.1 ng mL^−1^−100 ng mL^−1^ LOD: 5.6 pg mL^−1^	Clinical serum samples	[30]
GCE	Multifunctional zwitterionic peptide (anchoring, supporting, and antifouling domains)/electrode modifier	Aptasensor based on a direct affinity reaction	DPV ([Fe(CN)_6_]^3−/4−^)	TC	L.R.: 0.01–100 ng mL^−1^ LOD = 2.91 pg mL^−1^	Spiked milk sample	[40]
GCE	Multifunctional peptide with anchoring, antifouling, and recognition functions/biorecognition element	Direct affinity reaction at a GCE modified with the hierarchical *β*-Bi_2_O_3_−Au microsphere and the multidomain peptide	DPV ([Fe(CN)_6_]^3−/4−^)	Van	L.R.: 0.1−100 ng mL^−1^ LOD: 0.038 ng mL^−1^	Spiked milk, milk powder, and honey samples	[41]
AuE	Multifunctional amphiphilic peptides feature both a recognition and an aggregation (self-assembly) region/detector bioreceptorfor signal enhancement	Sandwich assay at a AuE modified with a cysteine-containing capture peptide andan amphipathic peptide labelled with Fc to perform an AISA strategy	SWV (Fc)	S100B (Melanoma)	L.R.: 0.2 nM–12.8 nM LOD: 0.02 nM	Spiked serum samples	[42]
PDA/HOF-101/ZnO ternary photoelectrode	BZP−cDNA conjugate formed through a click reaction/the BZP domain created an antifouling biointerface.	Aptasensor based on a direct affinity reaction coupled to the BZP	PEC	MUC1	L.R.: 50 fg mL^−1^–10 ng/mL LOD: 12 fg mL^−1^	Spiked human serum samples from healthy individuals	[43]
GCE	Multifunctional peptide/self-assembly, linker, and antifouling	Aptasensor at peptide nanoparticles-PANI-GCEs	DPV	CEA	L.R.: 1.0 pg mL^−1^–1.0 μg mL^−1^ LOD: 0.4 pg mL^−1^	Healthy and colon cancer human samples	[26]

*ACE2* angiotensin-converting enzyme 2, *AISA* aggregation-induced signal amplification, *AuNRs* gold nanorods, *AuNPs* gold nanoparticles, *BZP* branched zwitterionic peptide, *cDNA* complementary DNA, *CEA* carcinoembryonic antigen, *CFU* colony-forming units, *C-g-C*_*3*_*N*_*4*_ carbon self-doped graphitic carbon nitride, *CV* cyclic voltammetry, *DPV* differential pulse voltammetry, *D.R.* dynamic range, *EDC* 1-ethyl-3-dimethylaminopropyl carbodiimide, *EIS* electrochemical impedance spectroscopy, *Fc* ferrocene, *GCE* glassy carbon electrode, *hHBsAg* human hepatitis B surface antigen, *HOF* hydrogen-bonded organic framework, *LOD* limit of detection, *L.R.* linear range, *MB* methylene blue, *MCEPy *bipyridinium molecule, *MUC1* mucin-1, *NHS* N-hydroxy succinimide, *OPs* organophosphorus pesticides, *PAMAM* poly(amidoamine), *PANI* polyaniline, *PDA* polydopamine, *pDNA* probe DNA, *PEC* photoelectrochemistry, *PEDOT* poly(3,4-ethylenedioxythiophene), *PEG *poly(ethylene glycol), *PNP* p-nitrophenol, *PSA* prostate-specific antigen, *PWEs* paper working electrode, *RBD *receptor-binding domain, *SWV* square wave voltammetry, *TC* tetracycline, *UCNPs* core-shell-structured upconversion nanophosphors, *Van* vancomycin

**Table 2:** Electrochemical biosensing methods reported since 2022 using phage probes

Electrode	Biomimetic material/function	Fundamentals	Detection technique	Target analyte	Analytical characteristics	Application	Ref.
AuE	Phage L66/biorecognition element	Direct affinity reaction at a AuE/AuNPs/MPA/Phage L66	EIS ([Fe(CN)_6_]^3−/4−^)	*Salmonella*	L.R.: 20–2.0 × 10^7^ CFU mL^−1^ LOD: 13 CFU mL^−1^	Spiked milk, eggs, and chicken	[47]
SPCEs	Genetically engineered bacteriophage T7 encoding with *phoA* gene/biorecognition element	Monitoring of the ALP overexpression at a SWCNTs-SPCEs	DPV (1-NP)	*E. coli*	L.R.: 1–10^4^ CFU mL^−1^ LOD: 1.0 CFU mL^−1^	Inoculated spinach leaves	[48]
SPCE	Phage T4/biorecognition element	Direct affinity reaction at a SPCE/CNFs/Phage	EIS ([Fe(CN)_6_]^3−/4−^)	*E. coli*	L.R.: 10^2^–10^6^ CFU mL^−1^ LOD: 36 CFU mL^−1^	Spiked apple juice	[49]
GCE	Phage EP01/biorecognition element	Direct affinity reaction at a GCE/CFGO/CB/Phage	EIS ([Fe(CN)_6_]^3−/4−^)	*E. coli* O157:H7 GXEC-N07	L.R.: 10^2^–10^7^ CFU mL^−1^ LOD: 11.8 CFU mL^−1^	Spiked sterile milk and raw pork samples	[50]
SPCEs	Lytic phage ZCEC5 (*E. coli* T4-like virus)/biorecognition element	Direct affinity reaction at SPCEs nanostructured with AuNPs, MWCNTs, and WO_3_	EIS ([Fe(CN)_6_]^3−^)	*E. coli* O157:H7	L.R.: 10−10^4^ CFU mL^−1^ LOD: 3.0 CFU mL^−1^	Spiked food samples including beef meat, white cheese, tomato juice, tap water, and luncheon beef meat samples	[51]
GCE	M13 phage/biorecognition element	Direct recognition at a GCE/WS_2_QDs/AuNPs/phage	SWV ([Fe(CN)_6_]^4−^)	c-Met	L.R.: 1–1000 pg mL^−1^ LOD: 1.0 pg mL^−1^	Serum samples of CRC patients	[52]
GCE	Phage-encoded protein RBP 41/biorecognition element	Direct affinity reaction at a RBP 41/GO/AuNPs/GCE	DPV ([Fe(CN)_6_]^3−/4−^)	*Salmonella*	L.R.: 3.0−1.0 × 10^6^ CFU mL^−1^ LOD: 2.0 CFU mL^−1^	Inoculated food samples (milk and lettuce)	[53]
Au-SPE	M13 phage display affibody/detector bioreceptor for signal amplification	Sandwich immunoassay using cAb-MBs and phage display affibodies labelled with Ru(bpy)_3_^2+^ for signal amplification	ECL	Abrin	L.R.: 0.005–100 ng mL^−1^ LOD: 5 pg mL^−1^	Spiked milk, honey, and soil samples	[54]
Au-SPE	M13 phage display affibody/detector bioreceptor for signal amplification	Sandwich immunoassay using cAb-MBs and phage display affibodies labelled with Ru(bpy)_3_^2+^ and AuNPs@Ru(bpy)_3_^2+^ for signal amplification	ECL	Abrin	L.R.: 5 fg mL^−1^− 5 pg mL^−1^ LOD: 4.1 fg mL^−1^	Spiked rabbit plasma, milk, honey, and pollen	[55]
Au-SPE	Yamo I library phage display scFv/biorecognition element	Direct immunosensor by covalent immobilization of the scFv-ALP at a cysteamine modified Au-SPE	EIS ([Fe(CN)_6_]^3−/4−^)	Feline IgG	LOD: 10.42 nM	–	[56]
AuE	M13KO7 phage display VHH/biorecognition element	Direct immunosensor by covalent immobilization of the VHH at an MPA/MUA modified AuNPs-AuE	DPV ([Fe(CN)_6_]^3−/4−^)	VEGF	L.R.: 0.03–10,000 pg mL^−1^ LOD: 11 fg mL^−1^	Human serum from cancer patients	[57]
GCE	M13 phage display peptide/detector bioreceptor for signal amplification	Immunosensor fabricated at a GCE/*p*-NA/Ab and a phage display peptide labelled CdS NCs for signal amplification	SWV (Cd^2+^)	Molinate	LOD: 34 pg mL^−1^	Spiked river water samples	[58, 59]
AuE	M13 phage display peptide/biorecognition element	Direct affinity reaction at AuE/MUA/Peptide	DPV ([Fe(CN)_6_]^3−/4−^)	Ovomucoid	L.R.: 1.55–12.38 μg mL^−1^ LOD: 0.12 μg mL^−1^	Spiked egg and white wine samples	[60]
AuE	M13 phage display peptide/biorecognition element	Direct affinity reaction at a AuE/MPA/Peptide	EIS ([Fe(CN)_6_]^3−/4−^)	VEGF165	L.R.: 0.5–1000 pg mL^−1^ LOD: 0.15 pg mL^−1^	Human serum samples	[61]
AuE	M13 phage display peptide/biorecognition element	Direct affinity reaction at a AuE modified with AuNPs–MXene, streptavidin, and the biotinylated peptide	DPV ([Fe(CN)_6_]^3−/4−^)	Cathepsin B	L.R.: 3.9−125 nM LOD: 0.18 nM	Plasma/serum from Crohn’s patients	[62]
GCE	M31 phage display mimotope/competing tracer	Direct competitive assay at a GCE/NBCQDs@GO/Ab and enzymatic labelling using anti M13-HRP Ab	EIS ([Fe(CN)_6_]^3−/4−^)	*O,O*-dimethyl OPs	LOD: 0.003–0.014 ng mL^−1^	Spiked cucumber, cabbage, and lettuce	[63]
AuE	CX7C library phage display mimotope/biorecognition element	Indirect competitive assay at an AuE/MUA/phage using HRP/Ab@ZIF-8	DPV (TMB/H_2_O_2_)	TTX	L.R.: 1.0 × 10^−4^−1.0 × 10^3^ μg mL^−1^ LOD: 1.0 × 10^−5^ μg mL^−1^	Spiked puffer fish and Nassariidae samples	[64]

*Ab* antibody, *ALP* alkaline phosphatase, *AuE* gold electrode, *cAb* capture antibody, *CB* carbon black, *CFGO* carboxyl graphene oxide, *CFU *colony-forming units, *CNFs* carbon nanofibers, *CRC* colorectal cancer, *ECL* electrochemiluminescence, *GO* graphene oxide, *HRP* horseradish peroxidase, *MBs* magnetic beads, *MPA* 3-mercaptopropionic acid, *MUA* 11-mercaptoundecanoic acid, *MWCNTs* multiwalled carbon nanotubes, *NBCQDs@GO* nitrogen and boron-doped carbon quantum dots and graphene oxide, *NCs* nanocrystals, *1-NP* 1-naphthyl phosphate, *p-NA* p-nitroaniline, *QD* quantum dot, *RBP *receptor-binding protein, *scFv* single-chain variable fragment, *SPCE* screen-printed carbon electrode, *SPE* screen-printed electrode, *SWCNTs* single-walled carbon nanotubes, *TMB* 3,5,3’,5’-tetramethylbenzidine, *TTX* tetrodotoxin, *VEGF* vascular endothelial growth factor, *VHH* variable heavy homodimer or variable domain of heavy-chain antibody

**Table 3:** Versatility, advantages, and drawbacks inherent to peptide and phage probes in electrochemical biosensing

Probe type	Characteristics	Advantages	Disadvantages
Peptide probes	- Small size - High affinity - High stability - Structural and sequence diversity - Biocompatibility - Facile processability - Low immunogenicity - Easy obtaining with high yield - Affordable cost - Prone to modification with specific functional groups - Smaller binding regions than aptamers - Variable surface charges	- Automated chemical synthesis including modification with specific functional groups for immobilization or signaling - No need for laborious *in vivo* procedures and animal immunization - Higher chemical stability than antibodies - Feasible for the protease-based assays as natural substrates - Provide varied cross-linking methodology with the biosensing interface - Versatility of modification and use - Tuneable properties - Antibiofouling properties - Multifunctionality	- Relatively small size that might compromise the binding to biosensing elements - A high number of available peptides can make it difficult to choose and screen among them - The binding performance of peptide probes can vary from batch to batch and from the physical and chemical properties of the complex environment assayed (i.e., complex proteomes, ionic strength of the solutions, type of buffer, etc.)
Phage and phage receptor binding proteins	- Replicate only in living cells - Environmentally ubiquitous - Ecological - Safe to use (do not infect humans) - Genetically and chemically modified - Possibility to display peptides, - proteins, or antibody fragments - Natural affinity for their host bacteria - Small size - Insensitivity to proteases and anionic detergents - Ease of recombinant overexpression	- Controllable properties - Targeting biopolymers, toxins, proteins, or foodborne pathogens - Affordable - Easy to produce (they do not require inoculation or animal sacrifice) - Resistant to external factors (temperature, pH, ionic strength, organic/inorganic solvents, and proteases) - High sensitivity and specificity	- Relatively large size of whole-phage particles (avoided by using phage proteins) - Basal lytic activity (unless they are inactivated) may destroy target bacteria
Phage display receptors	- Express on their surface hybrid fusion proteins able to contain receptors (peptides, proteins, and antibody fragments)	- Simple screening, identification, and amplification of the phage of interest - Peptide, proteins, or antibodies are used specifically as receptors for binding to the target of interest - Determination of cytotoxins, herbicides, cancer markers, allergens, and animal immunoglobulins - Label-free bioassays	- Need to conjugate phage display receptors with multiple tag molecules to amplify the electrochemical response
Mimotopes	- Used for the determination of low molecular weight natural toxicants	- Avoid the problems associated with the preparation of complete competing antigens - Applied to the determination of mycotoxins, cholera toxin, pesticides, glycans, and infectious pathogens	- Difficult preparation of both mimetic peptides and antiidiotype antibodies - Failure rates in the biodisplay of mimotopes from the phage display peptide library - Complicate screening of antiidiotype antibodies from immunized animals - Mimotopes may have reduced affinity towards the primary antibody

## Biomimetic probes

The term “biomimetic,” derived from the Greek word biomimesis, composed of bios (life) and mimesis (imitate), was coined by Otto Schmitt in 1957 to designate design, adaptation, or derivation from Nature. It is used to name materials that do not occur naturally, which can be designed and synthesized by humans in the laboratory by imitating and/or overcoming the limitations of their biological counterparts [1, 2, 6].

The synthesis and application of biomimicking materials are in constant evolution fueled by advances in different areas (computational chemistry, combinatorial chemistry, phage display, etc.). Such materials include, among others, engineered proteins, cells and phages, peptides, carbohydrates, molecularly imprinted polymers (MIPs), supramolecular receptors, aptamers, recombinant antibodies, nanozymes, peptides, and oligonucleotides (locked nucleic acids, LNAs, peptide nucleic acids, PNAs, molecular beacons, DNAzymes, etc.). They are characterized by excellent physicochemical stability, durability, ease of storage, and affordability, compared to their natural counterparts and can be tailored to play different roles in a wide range of applications [1, 2].

One of the fields that has benefited greatly from these attractive and versatile materials is biosensing, an area in which they can function as recognition, signaling, and response amplification elements, antifouling materials, bionanomaterials, nanoscaffolds, artificial enzymes, enzyme substrates, etc.

Among all biomimetic materials, peptide and phage probes are considered nowadays star biomimetic functional materials capable of imparting tremendous opportunities in biosensing and particularly in electrochemical biosensing, helping to overcome some of the barriers that hindered the translation of this type of biosensing and/or devices to the real world.

## Modern electrochemical biosensing strategies

Biosensing and, in particular, electrochemical biosensing continue to consolidate as promising bioanalytical tools, at the forefront of modern detection techniques to satisfy the demands imposed by modern analysis in terms of realization by any user and in any environment, in a non-destructive, fast, and sustainable manner and with the aim of providing the most complete snapshot possible considering the large number of variables. Decisive for this has been the unique mix of attributes, intrinsic or acquired by alliances with other materials, strategies, and technologies, that accredit electrochemical biosensing: high selectivity and sensitivity, ease of use and low cost, fast response, suitability to analyze complex, turbid, and/or colored samples both at the multiplexed and multiomics level and its compatibility with simple and inexpensive instrumentation suitable for in-field and point-of-care readout devices that can be handled by any user in any environment, including remote and resource-limited settings [7].

The great advances demonstrated by electrochemical biosensors in recent years have gone hand in hand, among many other things, with the development of new electrochemical substrates, attractive surface chemistries, bioassay formats and amplification strategies, and the production and exploitation of new (nano)materials and bioreceptors [7, 8].

Although electrochemical biosensing technology can boast about having developed and/or having the potential to develop devices capable of facing pioneering applications of great relevance in different fields, there are still many and complex challenges to face to unfold its full potential and facilitate their presence outside the research environments. Among them, we can mention the improvement in sensitivity, reproducibility, and stability of the resulting biodevices, the simplification of their manufacturing and handling (reagentless [9], wash and calibration-free [10], one-pot and one-step operation [11], continuous real-time response [12] approaches), the discovery of new markers, the determination of toxic and small analytes, and the possibility to perform continuous analysis in fouling matrices. Fortunately, the developments derived from the coupling of electrochemical biosensing technology with the advances of biomimetic functional peptide and phage probes make us think that we are on the right track to successfully overcome some of the biggest challenges.

Enlightened by the above, this review article aims to offer the reader a critical overview of the latest advances and opportunities provided by bioinspired peptide and phage probes in electrochemical biosensing, with the purpose of giving the scientific community knowledge and arguments for trust more and more in this technology that, due to its brave and collaborative nature, continues to overcome barriers, considered tremendously challenging in biosensing (not only electrochemical), and stomping on new horizons.

Although the relevance, attractiveness, and topicality explain that other authors have also set their eyes on this topic and contributed with good review works on peptide-based [13–16], or bacteriophage-based [17–20] electrochemical biosensors, as far as we know there is no review that presents the state of the art and compares the exploitation of peptide and phage probes in electrochemical biosensing, which we consider very interesting due to the relevance of both types of probes and their complementarity and/or compatibility. This is addressed in this article by comprehensively presenting and discussing a selection of representative works from the last 2 years. At the end, critical and objective opinions are also provided on the challenges that must be faced, some more personal comparative reflections, and the promising perspectives of the topic.

## Breaking barriers in electrochemical biosensing with bioinspired phage and peptide probes

In this section, a timely and thorough coverage of the versatility and opportunities provided by peptide and phage probes in electrochemical biosensing based on selected reports mainly from the last 2 years is accomplished.

### Peptide probes

Peptides, short chain-like polymers containing less than 50 amino acids in length connected by peptide bonds, are star probes which have experienced an unstoppable boom in the development of electrochemical biosensing strategies with improved performance [7, 13–16, 20, 21]. Their use as probes in electrochemical biosensing is advantageous due to their small size, high affinity, stability, structural and sequence diversity, biocompatibility, facile processability, and lower immunogenicity compared with antibodies. They can be easily obtained with high yield and affordable cost as well as modified with specific functional groups for immobilization or signaling through automated chemical synthesis, avoiding the need for laborious *in vivo* procedures and animal immunization to reduce the use of laboratory animals and follow the EU recommendations on animal protection and replacement of animal-derived antibodies by non-animal-derived ones [22], and displaying higher chemical stability than antibodies [13, 23–25]. On the other hand, compared with nucleic acid aptamers, peptides have smaller binding regions and variable surface charges and are feasible for protease-based assays as natural substrates. Moreover, peptides provide a varied cross-linking methodology with the biosensing interface. For example, they can be immobilized on a gold surface through Au–S bonding using the cysteine thiol group, or they can be covalently immobilized by binding to carboxyl/amino group-functionalized interfaces through carbodiimide/succinimide chemistry [15, 16, 26].

Due to their versatility of modification and use, flexible variability, tuneable properties, and multifunctionality, peptides and their derivatives (complexes [27, 28], hydrogels [29, 30], nanotubes [15], nanoparticles [26], etc.) have been used in electrochemical biosensing as [7, 13–16, 31]:

- Interfacial materials (electrode modifiers) or self-assembled units/nanostructures (to immobilize other receptors in a suitable arrangement) to impart particular properties (antibiofouling, biocompatibility) and/or improve the biosensing performances;
- Recognition ligands to interrogate a wide variety of analytes;
- Enzymatic substrates (e.g., proteases and kinases);
- Enzyme mimics; and
- Signaling elements/carriers.

Due to their distinguished properties, antibiofouling, multifunctional, multimeric, and switching, peptides have gained special importance in recent years in electrochemical biosensing. Table 1 summarizes representative examples of methods developed during the last 2 years.

As can be deduced from Table 1, peptides have been exploited primarily as recognition elements [32, 33, 35, 37, 41], electrode modifiers [24, 26, 30, 39, 40], modifiers of other probes [34, 43], enzymatic substrates [36], mimicked enzymes [27, 28], and tracers [38, 42] for the electrochemical biosensing of a wide variety of targets including foodborne pathogens [32], immunoglobulins [33, 39], viral antigens [24, 38], cells [35], tumor markers [26, 27, 30, 34, 36, 37, 42, 43], pesticides [28], and antibiotics [40, 41].

In general, although the affinity of avidin/streptavidin for biotin has also been used for their attachment [34, 36], peptides have been immobilized on gold or nanostructured with AuNPs electrode surfaces through their self-assembly profiting the gold-thiol chemistry [24, 30, 33, 35, 37, 39, 40, 42]. In the photoelectrochemical (PEC) platform reported by Yin et al. [32], an antimicrobial peptide was immobilized on a flexible paper substrate modified with core-shell-structured upconversion nanophosphors ((UCNPs)@SiO_2_@Ag) and carbon self-doped graphitic carbon nitride (C-g-C_3_N_4_). A different approach has recently been described by Chen et al. [43], who fabricated a ternary photoelectrode by modifying a hydrogen-bonded organic framework (HOF-101) and polydopamine (PDA) onto a ZnO array electrode where a branched zwitterionic peptide (BZP) linked to complementary DNA (cDNA) through a click reaction was anchored.

Particularly relevant is the use of peptides as electrode modifiers [24, 26, 30, 39, 40], conjugated with other recognizing probes [34, 43], or as multifunctional bioreceptors [33, 35, 41] to implement fouling-free electrochemical biosensing strategies.

**Fig. 2:**
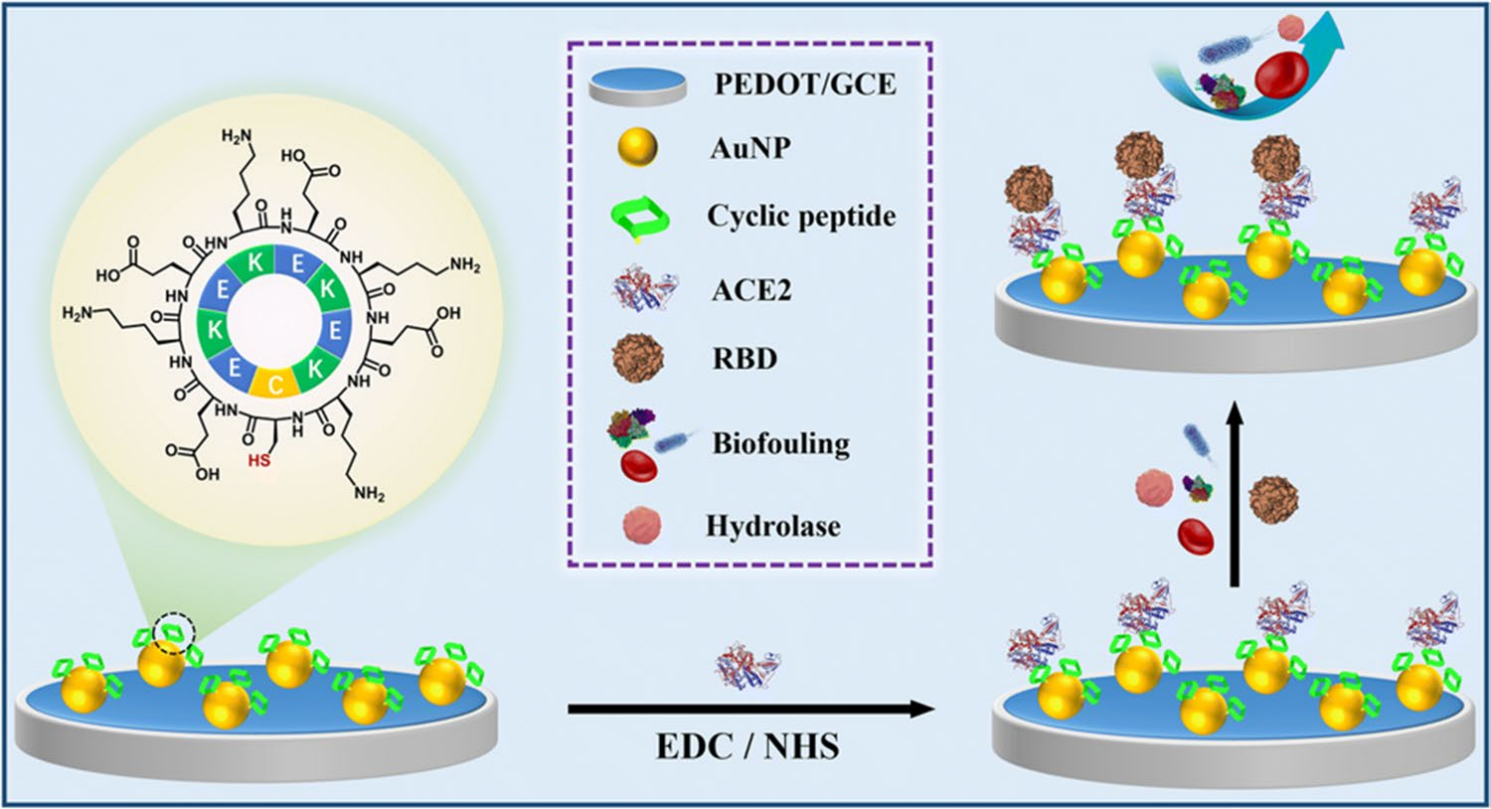
Biosensor for the determination of RBD of SARS-CoV-2 spike glycoprotein exploiting the use of a cyclic peptide as an electrode modifier. Reproduced from [24] with permission from the Royal Society of Chemistry

Peptides have been used as electrode modifiers in the development of affinity biosensors [24], immunosensors [30, 39], and aptasensors [26, 40, 43] with antifouling properties which have been applied to the determination of SARS-CoV-2 receptor-binding domain (RBD), IgM, PSA, TC, mucin-1 (MUC1), and carcinoembryonic antigen (CEA) in milk, blood, and serum samples. Among these peptides, zwitterionic peptides [26, 30, 40, 43], those involving D-amino acids [39], and cyclic peptides [24] stand out, the last two types showing an outstanding proteolytic resistance, thus overcoming one of the main complications faced by the proper functioning of peptide biosensors in complex environments [39]. For example, Han et al*.* [24] recently proposed a biosensor for the determination of the RBD of the SARS-CoV-2 spike glycoprotein by modifying a GCE/PEDOT/AuNPs with a cyclic peptide to impart self-fouling properties to the surface, and angiotensin-converting enzyme 2 (ACE2) as a target recognition element (Fig. 2). Due to the stable structure of the designed cyclic peptide and the absence of any N- or C-terminal amino acids, this biosensor exhibited noticeable resistance to biofouling and enzymatic hydrolysis even in human blood, thus enabling the accurate determination of the target in this complex matrix.

**Fig. 3:**
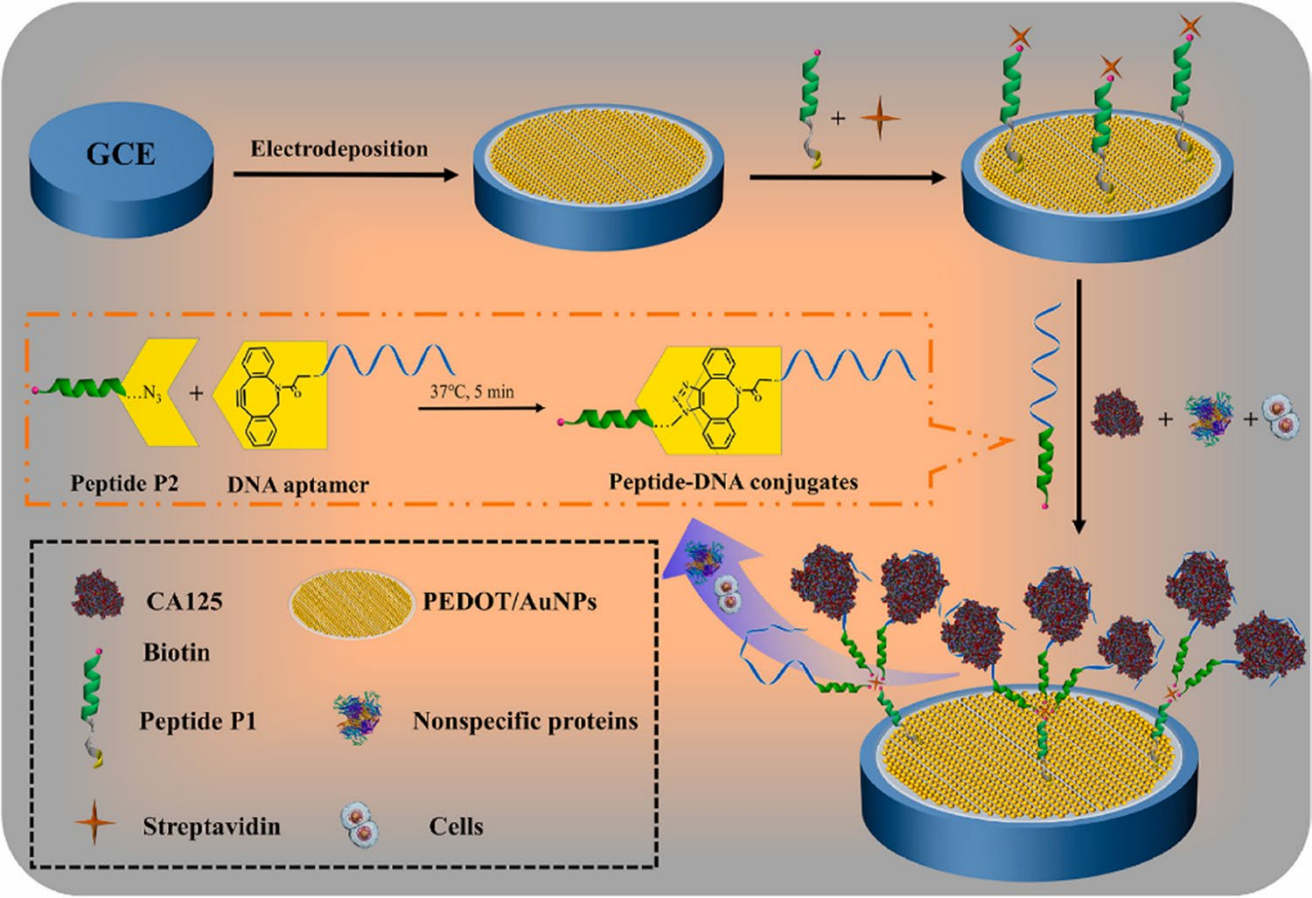
Electrochemical biosensor developed for the determination of CA125 involving the use of antifouling peptide-DNA conjugates. Reprinted from [34] with permission from Elsevier

It is worth drawing attention to the low fouling and highly sensitive electrochemical biosensor reported by Chen et al*.* [34] for the determination of CA125 involving antifouling peptide-DNA conjugates formed through a reagent-free click reaction (Fig. 3). The biosensor was able to analyze CA125 in undiluted human serum and provided a universal strategy to prepare antifouling biosensors through the conjugation of the antifouling peptides with the specific DNA probes. In addition, a new PEC aptasensor was developed recently and applied to the analysis of MUC1 in human serum [43].

**Fig. 4:**
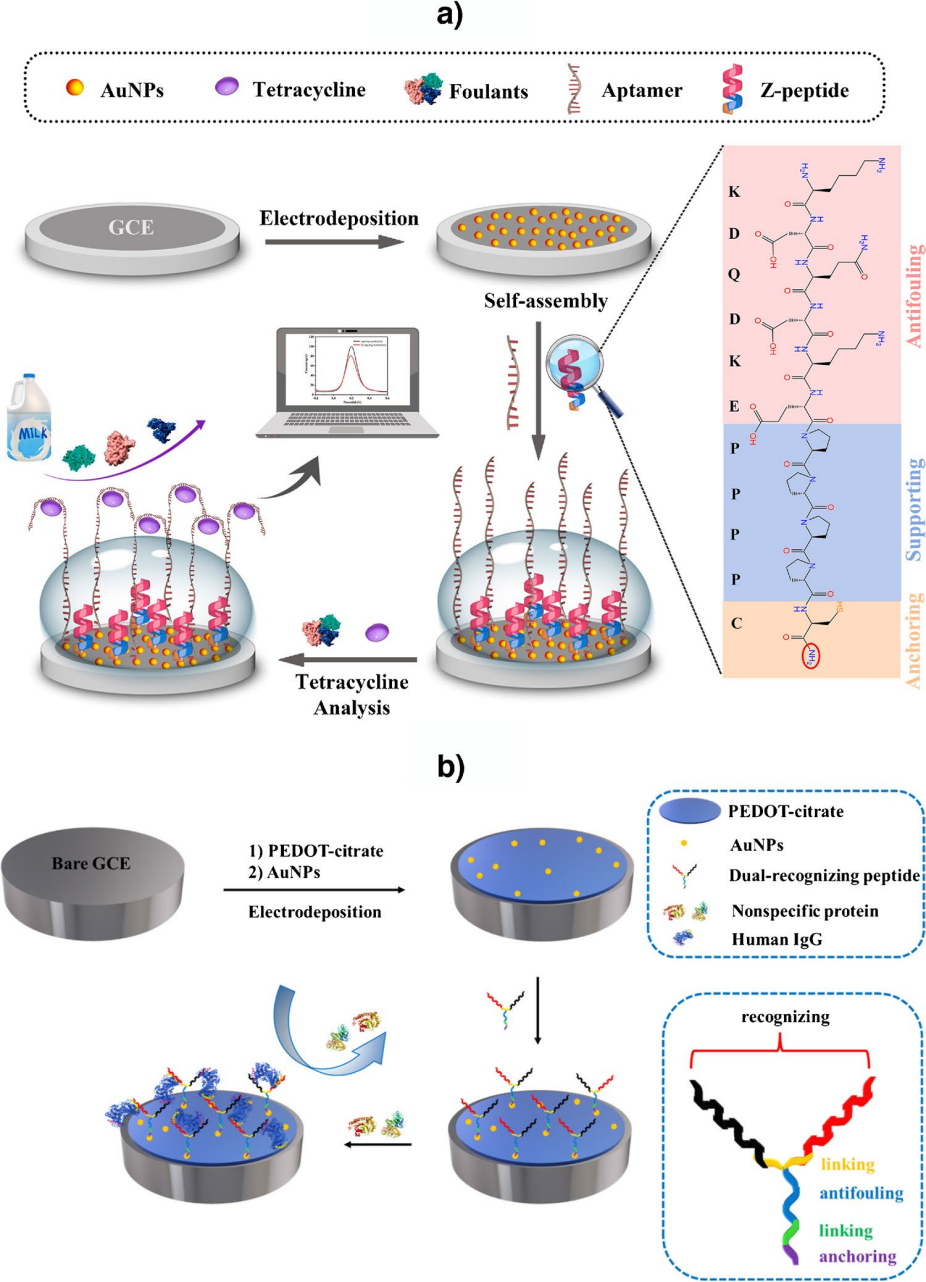
Examples of electrochemical biosensors involving multifunctional peptides as **a** electrode modifiers and **b** bioreceptors. Reprinted from **a** [40] and **b** [33] with permission from Elsevier

Multifunctional peptides (or “all-in-one” peptides) have different domains with different functions and have been exploited in electrochemical biosensing as electrode modifiers (Fig. 4a) [40, 41], bioreceptors (Fig. 4b) [33, 35], enzyme substrates [36], and elements for signal amplification [42].

**Fig. 5:**
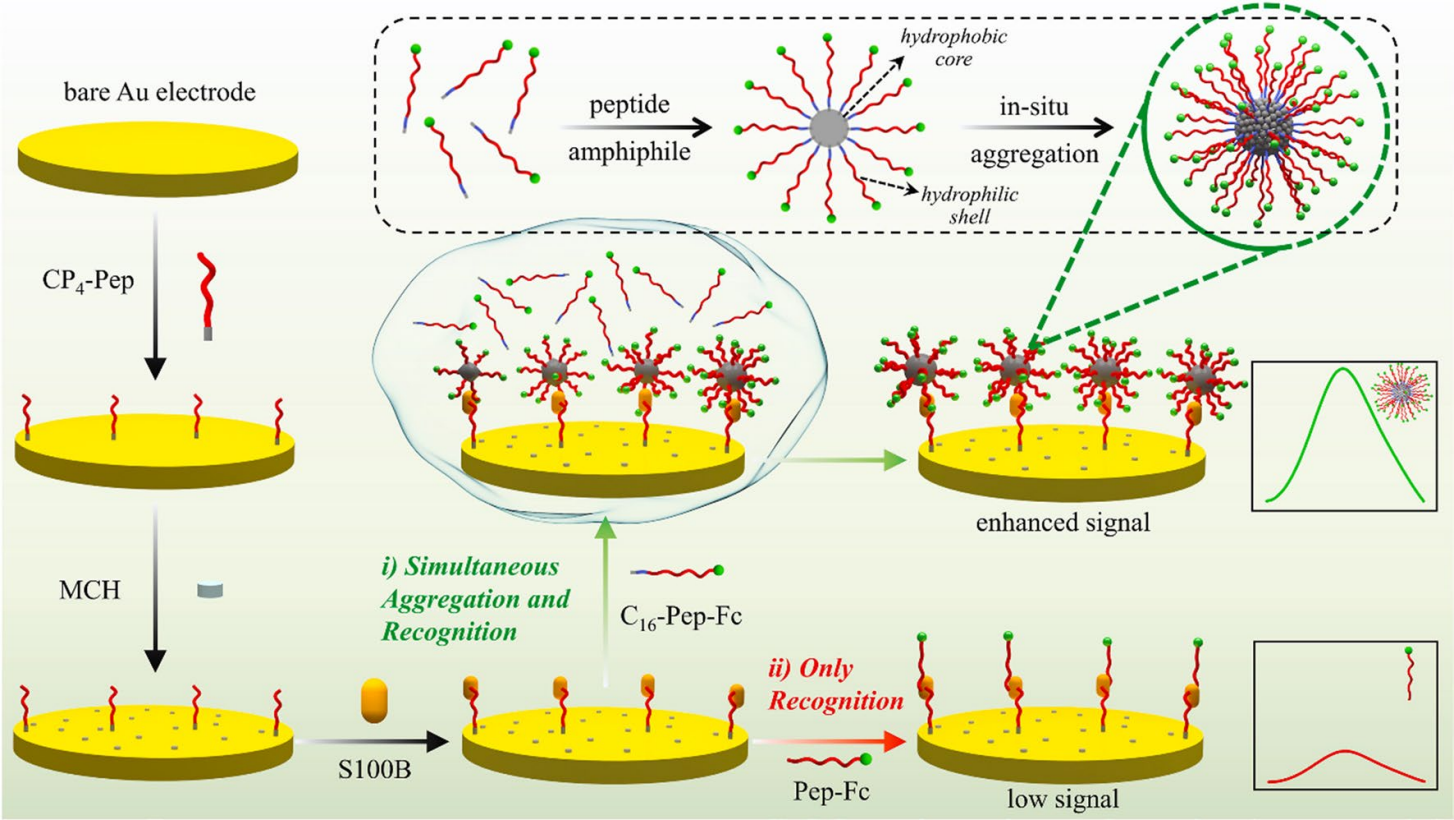
Use of multifunctional amphiphilic peptides in an AISA strategy for the preparation of a peptide-based biosensor for the determination of S100B. Reprinted from [42] with permission from Elsevier

Among the methods using multifunctional peptides, it is worth mentioning the peptide reported by Li et al*.* [33] which, in addition to anchoring and antifouling domains, possessed two recognizing branches to improve the target recognition efficiency and sensitivity (Fig 4b). Moreover, Yuan et al*.* [42] have employed multifunctional amphiphilic peptides in a sandwich assay to determine the melanoma circulating biomarker S100B. As can be seen in Fig. 5, the designed multifunctional amphiphilic peptides (C_16_-Pep-Fc) featured both a recognition region for the target and an aggregation (self-assembly) region for the formation, under mild conditions, of peptide nanomicelles in which the C_16_ tails were encapsulated within the hydrophobic core of the aggregates, and the relatively hydrophilic recognition fragment Pep and ferrocene (Fc) tag were exposed on the outer surface for S100B recognition and signal output. According to the authors, this aggregation-induced signal amplification (AISA) strategy provided a remarkable accumulation of electroactive Fc moieties achieving a LOD 10 times lower than the un-amplified approach.

**Fig. 6:**
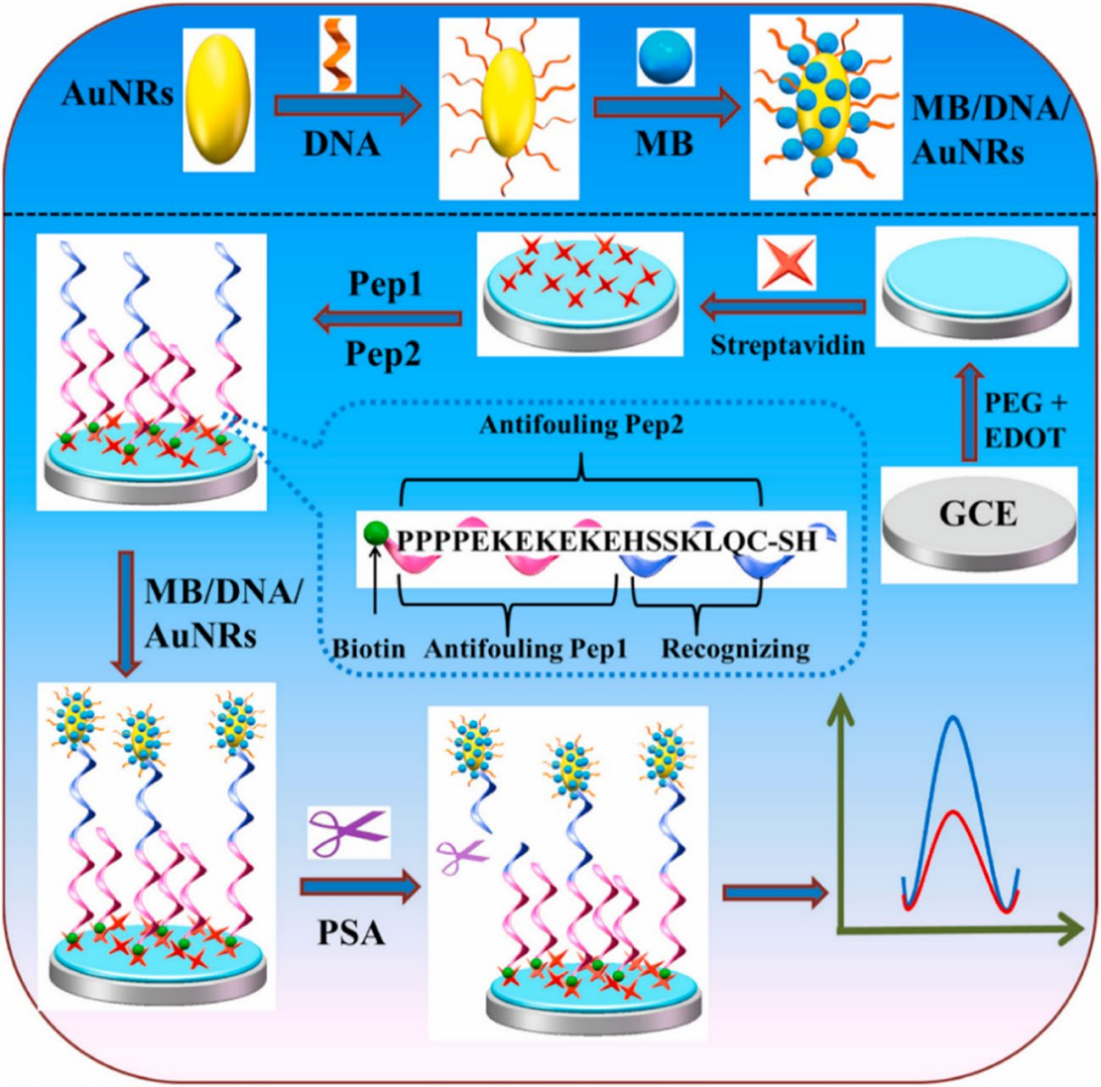
Electrochemical biosensor developed for the determination of PSA using a multifunctional peptide as enzyme substrate and a signal amplification strategy with MB/DNA/AuNRs. Reprinted from [36] with permission from Elsevier

Strategies, such as that described by Hui et al*.* [36], used multifunctional peptides as enzyme substrates in connection with signal amplification involving methylene blue (MB)/DNA/gold nanorods (AuNRs). In the presence of the target analyte (PSA), the multifunctional peptide is cleaved, releasing the MB/DNA/AuNRs immobilized on its terminal thiol group (Fig. 6). Monitoring the MB response by differential pulse voltammetry (DPV), the peptide-based biosensor achieved an LOD of 0.035 pg mL^−1^ and was employed for the analysis of undiluted serum.

Song et al*.* [26] designed a peptide with hydrophobic, linker, and antifouling differentiated regions that were self-assembled to peptide nanoparticles and applied in the construction of an electrochemical aptasensor. These peptide nanoparticles stood out not only for their antifouling capabilities but also for their enhanced stability in complex biological media.

**Fig. 7:**
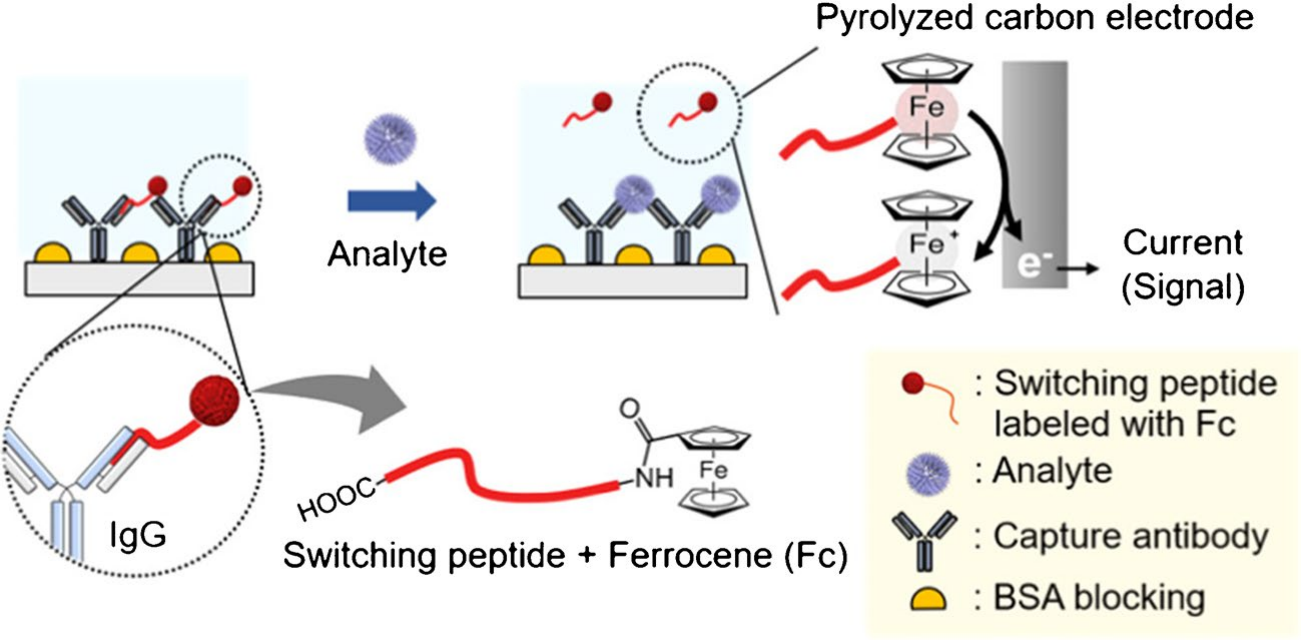
Immunosensor using an Fc-labeled switching peptide for the determination of hHBsAg. Reprinted with permission from [38]. Copyright 2022 American Chemical Society

Another less-used strategy for electrochemical biosensing involves peptide switching, designed to bind reversibly to the binding pocket of antibodies (IgG) by interacting with frame regions (FRs). Exploiting the use of these peptides, Park et al*.* [38] prepared a one-step immunosensor for the determination of human hepatitis B surface antigen (hHBsAg) which released the Fc-labeled switching peptide from the antibody in the presence of the target antigen and monitored the Fc response by DPV (Fig. 7).

**Fig. 8:**
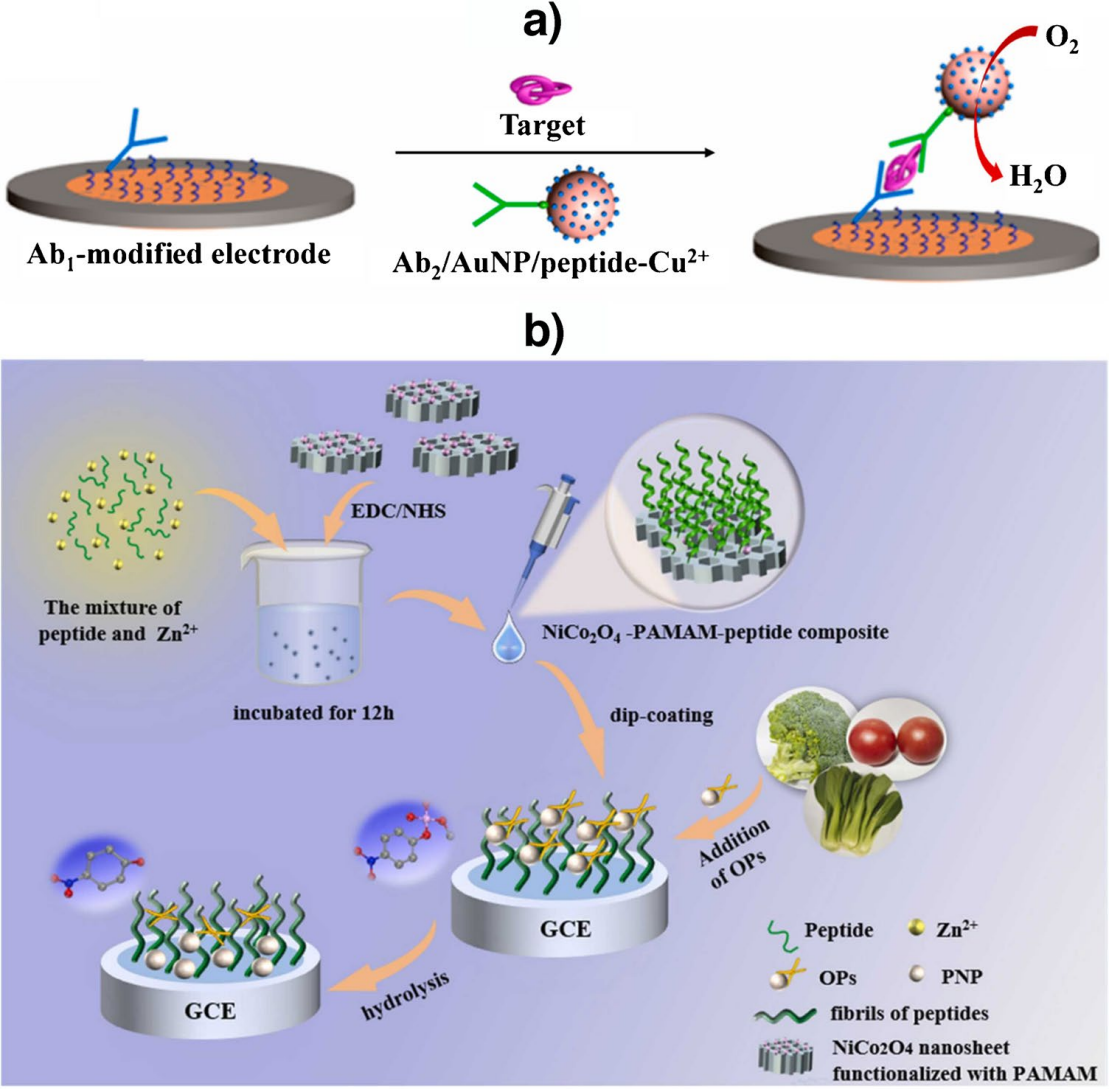
Use of peptide-cation complexes with **a** oxidase and **b** metalloenzyme mimicked activity as non-enzymatic tracers in **a** a sandwich-type immunosensor for the determination of PSA and **b** a peptide biosensor for the determination of OPs. Reproduced from a) [27] and b) [28] with permission from Elsevier

The use of metal-peptide complexes with mimicked enzymatic activity in both immunoassays and peptide bioassays should also be highlighted. For example, a sandwich immunosensor has been developed using AuNPs/peptide-Cu^2+^ conjugates as non-enzymatic tracers for the determination of PSA profiting the electrocatalytic reduction of oxygen by peptide-Cu^2+^ complexes monitored by DPV (Fig. 8a) [27]. Moreover, Yang et al*.* [28] profited the highly efficient metalloenzyme mimics of Zn^2+^-bonding peptides covalently immobilized on a NiCo_2_O_4_-PAMAM composite used as a modifier of a GCE for the determination of organophosphorus pesticides (OPs) monitored through SWV of *p*-nitrophenol (PNP) generated after their hydrolysis (Fig. 8b).

### Phage probes

Recently, we have witnessed the exploitation of intact bacteriophages (or phages), phage proteins (phage-encoded proteins or receptor-binding proteins, RBPs), and their derivatives (phage display peptides, affibodies, single-chain fragment variable (scFv) antibodies, variable heavy homodimer (VHH), or mimotopes) as attractive alternative bioprobes for electrochemical biosensing [18, 44–46]. Table 2 summarizes the rationale and relevant characteristics of representative examples of electroanalytical methods reported in the last two years involving the use of bacteriophage-based bioprobes.

#### Phages and phage receptor-binding proteins

**Fig. 9:**
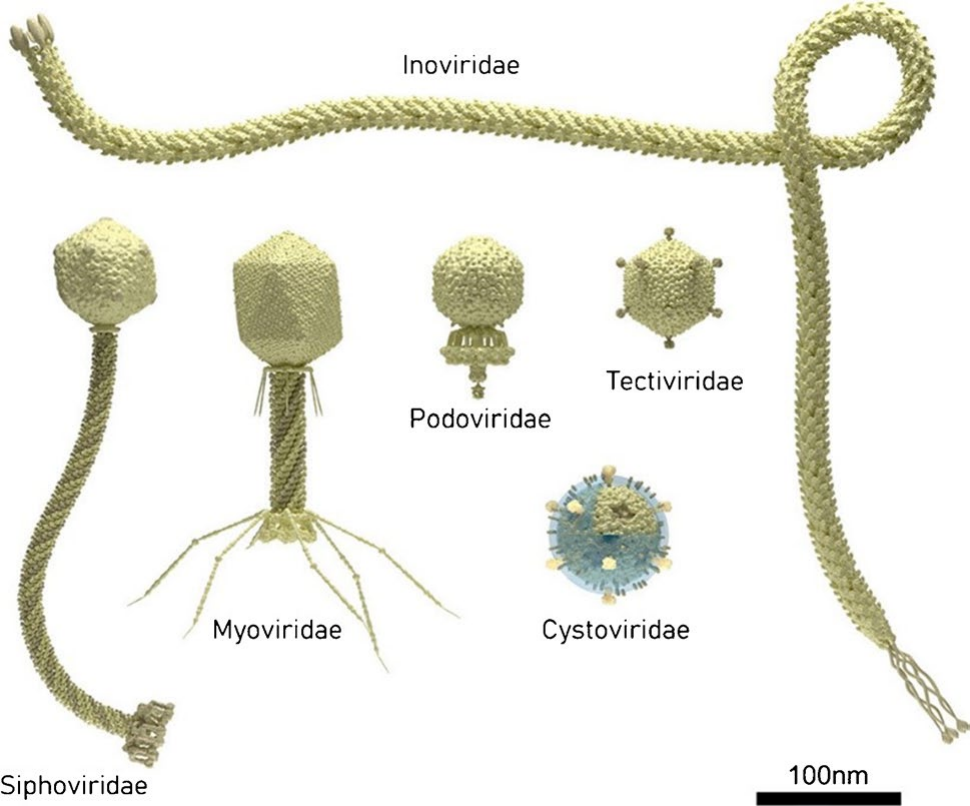
Bacteriophage structures more frequently reported in the literature on phage functionalization. Reprinted with permission from [66]. Copyright 2021 American Chemical Society

Phages are extraordinarily robust and stable virus particles that lack their own metabolic machinery and specifically target and infect bacteria for their replication [18, 44, 53]. In addition to their high specificity, phages only replicate in living cells and are environmentally ubiquitous, ecological, and safe to use since they do not infect humans. Moreover, they can be genetically and chemically modified, making their properties controllable [65]. They specifically attach to the host bacteria via tail fibers and insert their genome (RNA or DNA) into the bacterial genetic material to initiate the replication of prophages, resulting in the production of mature virion particles. Multiplication and propagation of virions within bacteria proceed in two ways, namely, lytic cycle and lysogenic cycle [18]. Phage structure exhibits a broad range of variations, which can be categorized into a few standard forms (Fig. 9). A limited set of phage morphologies is overrepresented in the literature regarding phage immobilization as bioprobes. They include long contractile-tailed phages (*Myoviridae*, i.e., T4), long noncontractile-tailed phages (*Siphoviridae*), short-tailed phages (*Podoviridae*, i.e., T7 or P68), and filamentous phages (*Inoviridae*, i.e., M13 and fd). Phage families without tails, such as *Tectiviridae* (which includes non-tailed icosahedral phages like PRD1) and *Cystoviridae* (which features an outer lipid membrane and lacks a tail, such as phage phi6), are less commonly found in phage immobilization literature.

Phages are composed of a protein coat composed of different proteins that encapsulate their RNA or DNA genome, which includes four to hundreds of genes. Phage coat proteins can be conjugated or genetically modified to display peptides [23, 67], proteins, or antibody fragments targeting a wide variety of molecules, including biopolymers, toxins, proteins, or foodborne pathogens [3, 65, 68]. In comparison with other biological recognition elements such as antibodies or aptamers, phages are cheaper, very specific and easy to produce (they do not require inoculation or animal sacrifice), and more resistant to external factors (temperature, pH, ionic strength, organic/inorganic solvents, and proteases) [3, 4, 17, 18, 47, 49, 51, 52, 65, 69, 70].

Phages can be classified into three different categories according to their morphology, life cycle, and way of propagation [3, 4]:

- *Lytic or productive phages* (T3, T4, T7, and MS2) only capable of replicating their genome, assembling phage structured components, and releasing from bacteria after synthesis and cell death.
- *Temperate or lysogenic phages* (λ), which can multiply via a lytic cycle, as productive phages, or can incorporate their genome into the bacterial chromosome producing a quiescent state (prophage).
- *Filamentous phages* (f1, fd, or M13), which are lysogenic phages, characterized by their long rodlike shape, that do not lyse their host cell but secrete the newly assembled virions and continue the process.

It is precisely the natural affinity of phages for their host bacteria and their conserved structures that has allowed their widespread exploitation as bioreceptors in the development of electrochemical biosensing platforms for bacteria [51]. Lytic phages that cause the lysis of bacterial cells releasing endogenous components that act as analytes for detection have also been used [51]. Another promising approach is the use of reporter phages, genetically engineered to possess a reporter gene that encodes the expression of exogenous enzymes in the phage genome, such as *lux* and *lacZ* [48], activated when the phage interacts with the target bacteria [18, 19].

**Fig. 10:**
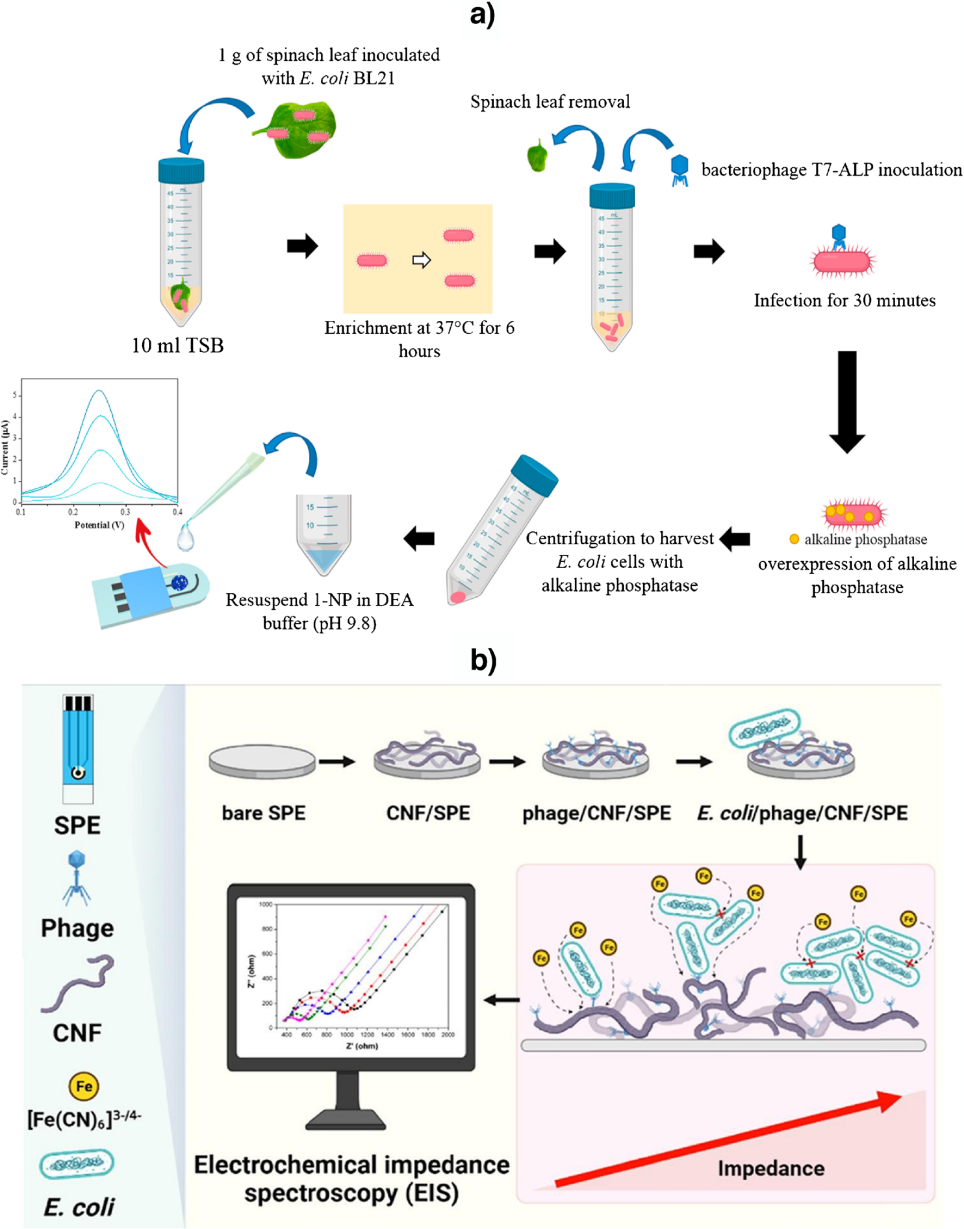
**a** Portable biosensor for the determination of *E. coli* based on the electrostatic immobilization of a phage on a SPE decorated with electrospun polyacrylonitrile (PAN)-based carbon nanofibers (CNFs) via drop-casting. **b** Electrochemical biosensing strategy based on the use of a genetically engineered bacteriophage that can trigger ALP overexpression in the presence of *E. coli*. Reprinted with permission from **a** [48] and **b** [49] with permission from Elsevier

As shown in Table 2, phages have recently been used as recognition elements in electrochemical biosensing mainly to detect specific bacteria, mostly *Escherichia coli* (*E. coli*) [48–51], due to the ease, speed, and cheap production of its phages [3], but also *Salmonella* [47]. Although much more rarely, phage-based biosensors have been used for the determination of carcinogenic markers [52]. These bioplatforms involve the covalent [47, 50, 51] or non-covalent (Fig. 10a) [49] immobilization of the corresponding phages on conventional or screen-printed electrode (SPE) surfaces nanostructured with different nanomaterials, and the monitoring of the affinity reaction using label-free strategies. The reported bacterial bioplatforms achieve LODs for *E. coli* or *Salmonella* between 1 and 36 CFU mL^−1^ and were utilized for their analysis in a wide variety of inoculated food samples. The bioplatform for the determination of the carcinogenic marker (c-Met) allows its detection at the pg mL^−1^ level and was successfully applied to the analysis of serum samples from CRC patients. Particularly relevant works are the biosensor developed by Abdelhamied et al*.* that uses lytic bacteriophage [51], and the method reported by El-Moghazy using a genetically engineered bacteriophage T7 encoding with *phoA* gene that can trigger alkaline phosphatase (ALP) overexpression in the presence of the target bacteria. The enzymatic hydrolysis of 1-NP was monitored by DPV (Fig. 10b) [48].

**Fig. 11:**
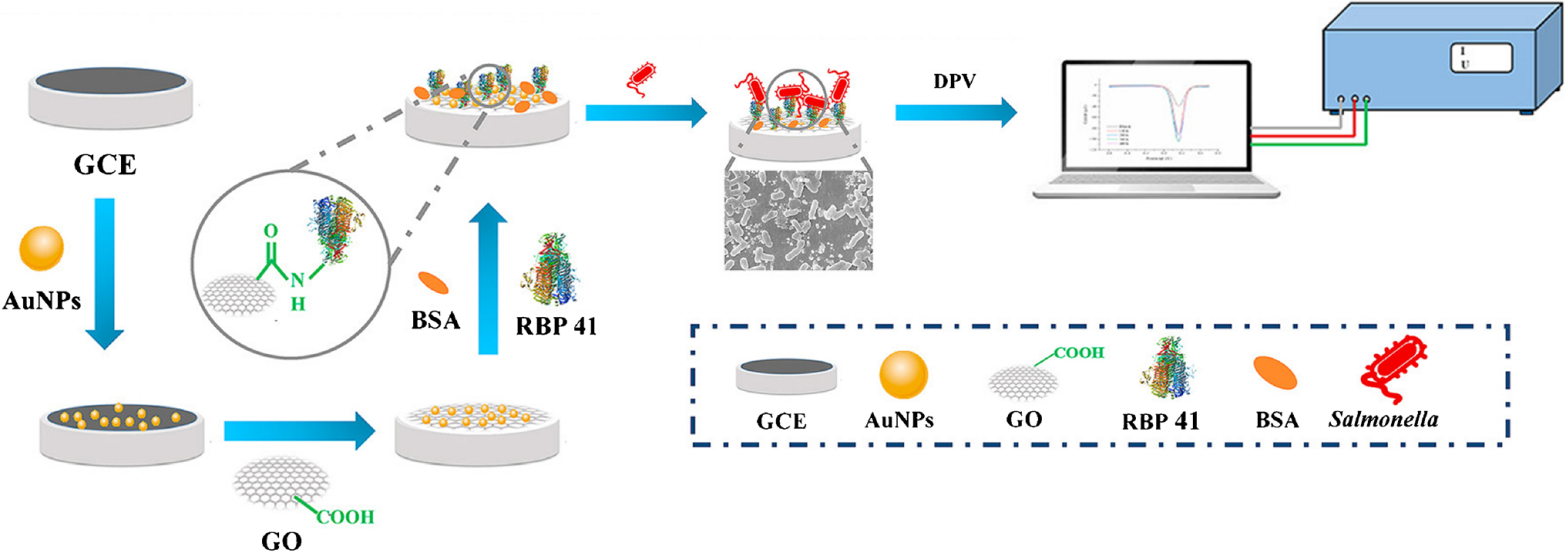
Electrochemical biosensor using the RBP 41 for the determination of *Salmonella*. Reproduced from [53] with permission from Elsevier

Since most phages interact with receptors on the bacterial cell surface through RBPs in their tails, such as tail fiber proteins, tail spike proteins, and baseplate proteins, RBPs have been used as bioreceptors [53]. RBPs are highly variable trimeric structures responsible for recognizing bacterial surface-specific receptors such as lipopolysaccharides and outer membrane proteins. They exhibit advantages due to their high sensitivity and specificity, small size, high stability to extreme pH and temperature, insensitivity to proteases and anionic detergents, and ease of recombinant overexpression. Indeed, the use of phage proteins instead of whole-phage bioprobes avoids the drawback of using the relatively large size whole-phage particles and their basal lytic activity (unless they are inactivated) that may destroy target bacteria [71]. The biosensor reported by Ding et al*.* exploited RBP 41 for the determination of *Salmonella* (Fig. 11) [53].

#### Phage display receptors

**Fig. 12:**
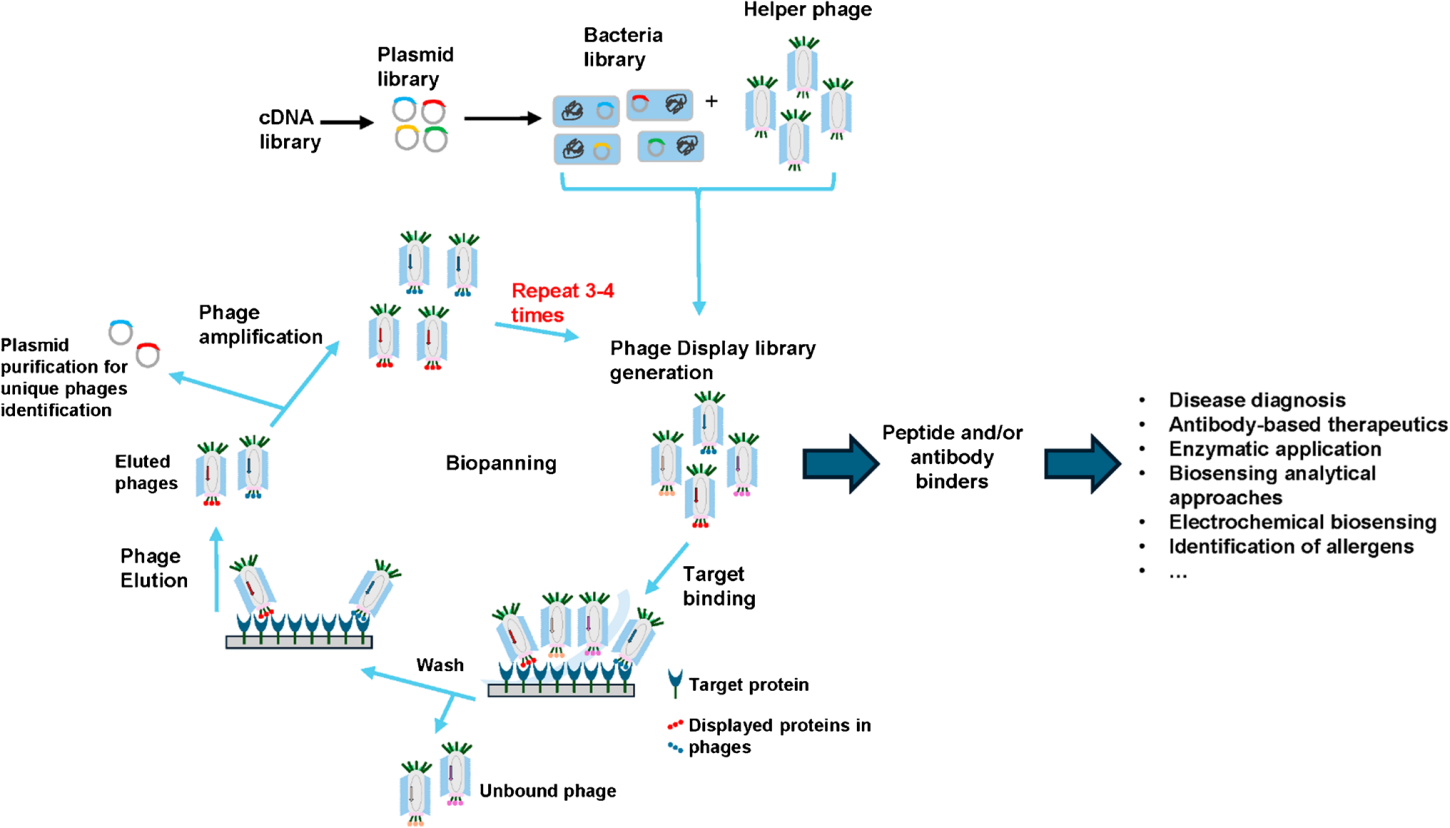
Scheme illustrating the phage display technology used to identify specific binders against the target of interest. Phage display involves a process called biopanning consisting of 3 or 4 cycles for the selection of the specific targets using mainly the M13 phage. The direct immobilization technique is depicted in the figure. Within bacteria, the amplification of phages takes place, with each eluted phage clone undergoing multiplication by a factor ranging between 10 and 100 times during each round of biopanning. Potential applications of peptide and/or antibody phage display are also highlighted in the figure

Phage display technology, developed by G. P. Smith in 1985 and reviewed by himself and V. A. Petrenko in 1997, allows genetically modifying bacteriophages so that they insert foreign DNA into the genes that encode their coat proteins [72, 73]. In this way, phages are released from the host cell and can express on their surface hybrid fusion proteins capable of containing receptors such as peptides, proteins, and antibody fragments (single-chain variable fragment, scFv and variable domain antibodies, VHH). The presence of these receptors simplifies the screening, identification, and amplification of the phages of interest in the complex population generated (phage display libraries, assemblies of about 10 billion of phage clones each harboring a different variant of the displayed entity) that are subsequently enriched infecting *E. coli* in a process that is globally known as biopanning [4, 65, 68, 73, 74]. Thus, phages have been largely used in the last years for phage display to identify peptide, proteins, or antibodies specifically as receptors for binding to the target of interest (Fig. 12). The M13 filamentous phage is the most widely used for peptide, proteins, or antibody phage display [68, 73].

In this way, phage display receptors consist of two modules of interest in bioanalytical applications, the one used for target recognition and the phage shell, with numerous protein copies and inherent functional groups for chemical modifications [73], which can be used as a carrier for the massive enrichment of signal molecules, making them very attractive tracers for signal amplification in high-sensitivity biosensors [54, 55].

Phage display is a constantly evolving flexible technology due to discoveries and innovations in chemical and molecular engineering and offers a new mean to discover recognition elements, even beyond natural and known biomolecular interaction.

As shown in Table 2, phage display peptides, affibodies, scFv, and VHH have been recently employed in the development of electrochemical bioplatforms for the determination of targets of very different nature including cytotoxins, herbicides, cancer markers, allergens, and animal immunoglobulins. These methods are all based on label-free bioassay, where phage display receptors have been used as capture or detection elements conjugated with multiple tag molecules to amplify the electrochemical response.

**Fig. 13:**
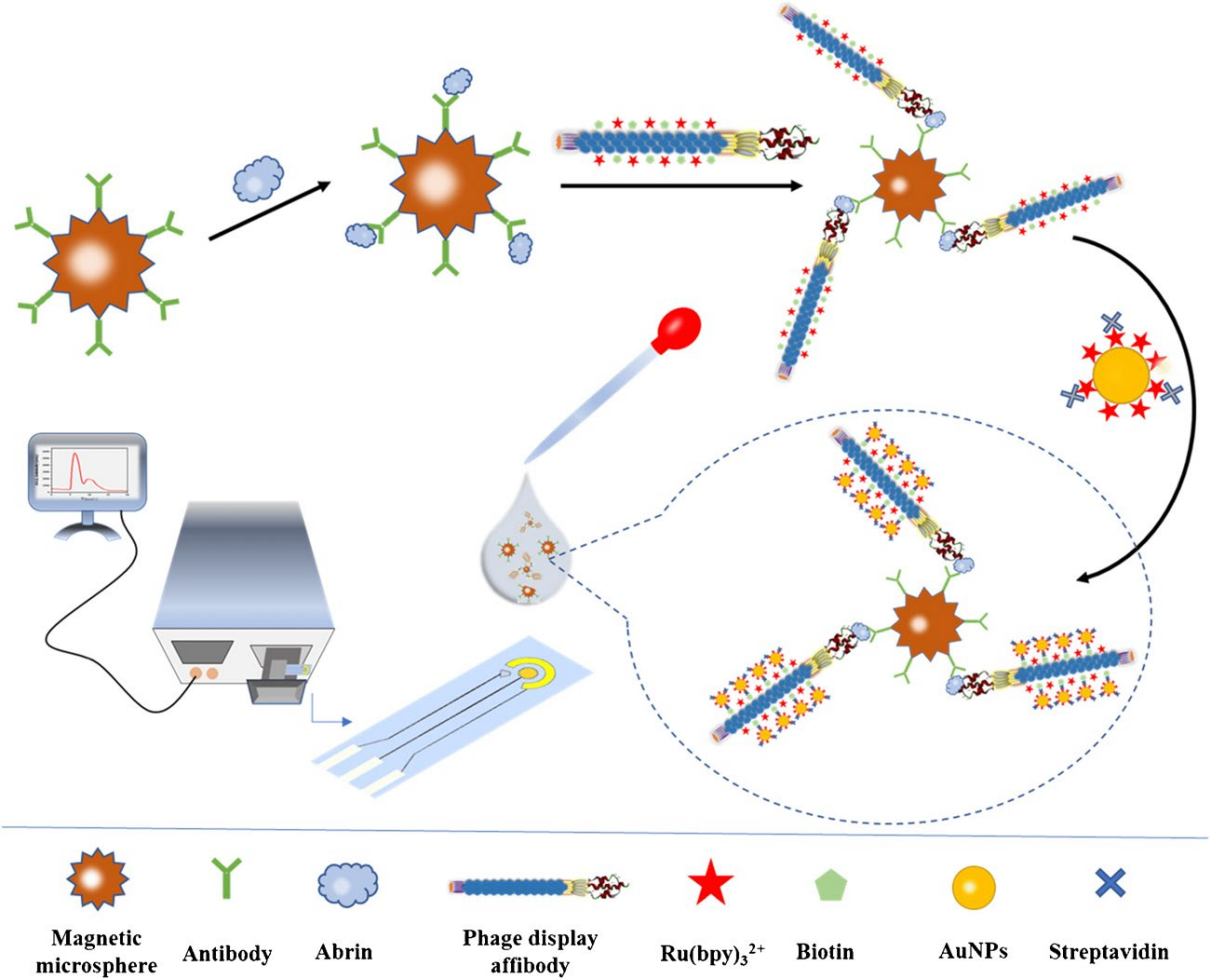
Sandwich electrochemical immunoassay for the determination of abrin using antibody-coated MBs and phage display affibodies labeled with Ru(bpy)_3_^2+^ and AuNPs@Ru(bpy)_3_^2+^. Reprinted from [55] with permission

Illustrative examples are sandwich immunoassay formats that combine the advantages provided by antibody-coated magnetic beads (MBs) [75] and phage display affibodies labeled with multiple Ru(bpy)_3_^2+^ tags for the sensitive determination of abrin by electrochemiluminescence (ECL) (Fig. 13) [54, 55].

Tocco et al*.* [58, 59] prepared immunosensors for the determination of molinate using a phage display peptide conjugated with CdS nanocrystals (NCs) for signal amplification and monitoring of Cd^2+^ ions by SWV.

Integrated bioplatforms have been developed by covalently immobilizing via EDC/NHS chemistry phage display scFv, VHH, and peptides on gold electrodes modified with self-assembled monolayers of alkanethiols [56, 57, 60, 61] or biotinylated phage display peptides on nanostructured gold electrodes modified with streptavidin [62]. These bioplatforms involved direct affinity formats and transduction by DPV or electrochemical impedance spectroscopy (EIS) in the presence of [Fe(CN)_6_]^3−/4−^, for the determination of feline IgG [56], vascular endothelial growth factor (VEGF) and its different isoforms [57, 61], ovomucoid [60], and cathepsin B [62]. They were applied to the analysis of supplemented food samples and serum/plasma of patients with cancer or Crohn’s disease.

#### Mimotopes

Mimotopes, or epitope mimics, are peptides or microproteins that mimic antigenic epitopes and can specifically bind to antibodies and compete with analytes for binding sites. Peptide mimotopes are potential antigens for the development of non-toxic and/or ecological assays and for the establishment of safe vaccination strategies [76]. To date, two types of mimotopes have been mostly described: mimetic peptides and antiidiotypic antibodies (Ab2) [74].

Mimetic peptides are produced by phage display technology using a primary antibody as a target, while the latter, which include monoclonal Ab2, polyclonal Ab2, and nano-Ab2, can be obtained by immunization of animals with the primary antibody. Generally, mimotopes are used for the determination of low molecular weight natural toxicants, such as mycotoxins, which require competitive formats, avoiding the problems derived from the preparation of complete competing antigens that involves complex procedures, long reaction periods, batch errors, significantly high costs, decreased antibody affinity, instability problems, cross-reactions, and potential safety threats to experimenters [5, 64, 74, 77].

For their application in immunoassays, mimotopes are functionalized, through chemical synthesis or molecular fusion techniques, with transport proteins or signaling elements, and used as coating antigens, as standard surrogates, or as competing tracers [74]. Although mimotopes have shown significant benefits in certain immunoassays, they still have some limitations. The preparation of both mimetic peptides and antiidiotype antibodies is difficult. Biodisplay of mimotopes from the phage display peptide library has always high failure rates, while screening of antiidiotype antibodies from immunized animals also presents great challenges. It is important to expand the diversity of the peptide library and improve the technology for detecting positive clones. Furthermore, to date, most mimotopes used in immunoassays have demonstrated similar or slightly superior performance in the assay. It is also important to highlight that in some cases the mimotopes have reduced affinity towards the primary antibody and that the use of directed mutagenesis could improve the characteristics of the mimotopes and the performance of the immunoassays [74].

**Fig. 14:**
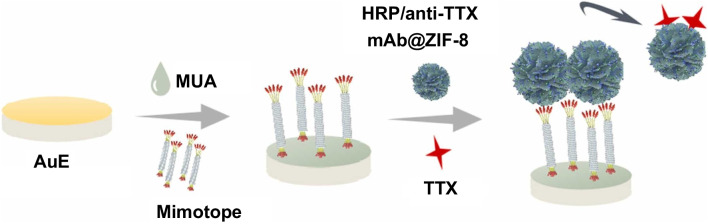
Indirect competitive immunosensor for the determination of tetrodotoxin (TTX) using a phage display mimotope. Reproduced from [64] with permission from Elsevier

To date, mimotopes have been used for the determination of mycotoxins [5, 64, 74, 78], cholera toxin [77], pesticides [63], glycans [76], and infectious pathogens [79], among others. As shown in Table 2, during the last 2 years, the use of mimotopes has been exploited in immunosensors constructed on nanostructured electrodes for the determination of pesticides [63] and mycotoxins (Fig. 14) [64]. These strategies used direct [63] or indirect [64] competitive methods and transduction with or without a label. They were successfully employed for the sensitive and selective determination of the targets and applied in the analysis in supplemented vegetables and fish samples.

### Take-up message, challenges, and perspectives

In addition to the simplicity and versatility of manufacturing and modification, and its greater stability compared to other natural or artificial probes (e.g., antibodies and aptamers), the exploitation of peptide and phage probes in electrochemical biosensing is decisive in reducing the list of limitations of this technology and increasing the opportunities for continually opening new and attractive horizons. Table 3 summarizes the pros and cons of peptide and phage probes for electrochemical biosensing.

The latter advances have put on the table the potential of using peptide probes to develop biosensing strategies with notable resistance to biofouling and enzymatic hydrolysis allowing continuous long-term monitoring even in human blood (cyclic or D-based amino acid peptides); considerably simplify its manufacturing (all-in-one peptides); improvements in sensitivity and selectivity (multimeric peptides or peptides responsible for response amplification strategies); and improvements in robustness under certain experimental conditions (metal-peptide complexes as enzyme mimics).

On the other hand, phage probes have found their niche in electrochemical biosensing to overcome the limitations represented by the determination of toxic and small analytes, the exploration of molecular interactions beyond those natural and known, the discovery of new molecular markers, and the development of devices with improved sensitivity and selectivity.

The reported works lead to suggest that certain opportunities should be better pursued with a certain type of probe, for example, the antifouling and antihydrolysis capabilities with peptides and the determination of toxic targets and the exploration of other interactions beyond natural and known with phages. However, the state of the art with myriad of exciting possibilities invites us to think that the two types of probes looked at in this review offer multifunctional properties and tremendous opportunities to continue exploring and improving the performance of electrochemical biosensing.

This is why we personally would set our future sights on multifunctional peptides (ideally also multimeric) and on receptors displayed in phages that are also multifunctional because they have both the recognition module and the phage module. In fact, phage display peptides can be considered an attractive hybrid that combines the advantages of both types of probes.

However, it is important to highlight that although the scenario is exciting, there is still a lot and very complex work to do. This technology must continue to advance as it has until now, through the design, preparation, and application of new peptide and phage probes that provide new opportunities and improved performance and stability. In addition, it must start interacting with other technologies on everyone’s mind these days, such as artificial intelligence, which has a lot to offer in this area, both in the modeling of probes with improved properties and in the processing of data to advance in universality and robustness of the technology overcoming the matrix effect and variability issues in complex samples, allowing recognition in real time. Enhance the multiplexed and/or multiomics character allowed by electrochemical biosensing, proper evaluation of reproducibility and long-term stability, test the developed bioplatforms robustness by applying to the analysis of endogenous contents in a sufficient number of samples, by different users and in different environments, promoting the use of sustainable electrode substrates (such as paper) and other electrochemical techniques apart from voltammetry and EIS, such as ECL and PEC, are other of the multiple tasks included in the extensive and complex roadmap currently being drawn. And all this with the purpose of transforming the corresponding bioassays from analytical proof of concepts to commercialized real-life solutions allowing that all of us, and other types of biosensing detection apart from electrochemical, can benefit from these important advances that occur uninterruptedly at the research level. Something that obviously must be achieved by making information flow, and through close and generous large-scale multidisciplinary collaboration between researchers, producers, end users, and society in general.

## Data Availability

Not applicable.
